# The kinetochore and the origin of eukaryotic chromosome segregation

**DOI:** 10.1073/pnas.1908067116

**Published:** 2019-06-07

**Authors:** Mark C. Field

**Affiliations:** ^a^School of Life Sciences, University of Dundee, Dundee DD1 5EH, United Kingdom;; ^b^Biology Centre, Institute of Parasitology, Czech Academy of Sciences, 370 05 České Budějovice, Czech Republic

All organisms must faithfully segregate their DNA during cell division to safeguard complete inheritance of the genome. In eukaryotes, mechanisms of cell and nuclear division are highly variable, and while these usually involve the use of a mitotic microtubule-based spindle and a kinetochore (KT) that physically links the chromatin and spindle, beyond this, the arrangement and manner in which mitosis is completed can adopt one of a vast number of disparate pathways (1, 2). Each of these pathways requires the participation of multiple cellular functions, including the nucleoskeleton, centromeres (chromatin-marked KT assembly sites), the nuclear envelope, and the nuclear pore complex (NPC), to achieve the ultimate goal of partitioning a complete genome to both daughter cells. Because the eukaryotic genome is contained on multiple DNA elements and is frequently diploid makes this task even more challenging. In PNAS, Tromer et al. (3) revisit the origin of a key component, the KT, using highly sensitive sequence and architectural search methods to provide a possible evolutionary history.

**Fig. 1.**
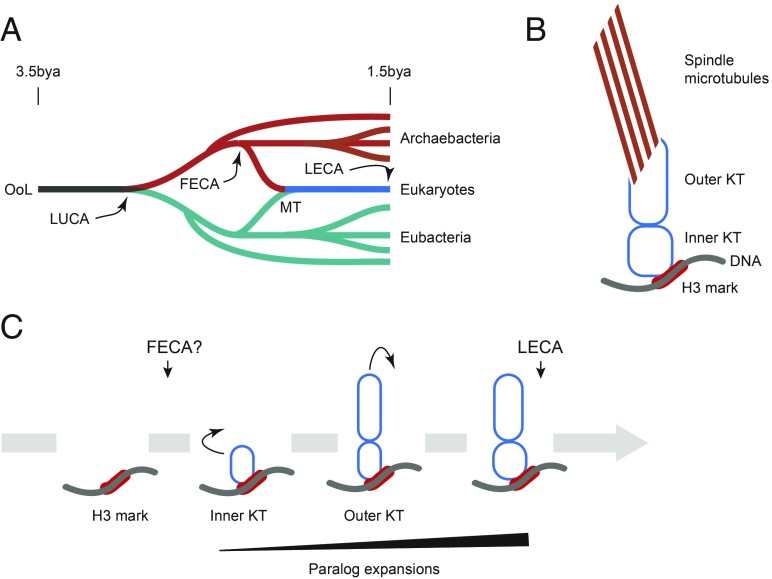
Evolution and origin of the KT. (*A*) Overview of the evolution of life on Earth, with emphasis on relationships between eukaryotes and prokaryotes. The origin of life on earth (OoL) is placed at about 3.5 billion years ago (bya). LUCA, last universal common ancestor. Red indicates Archaebacterial lineages, including that which led to the eukaryotes. FECA, by consensus, represents the final branching from the archaeal linage leading to eukaryotes; evidence indicates that this lineage resides within the “TACK/Asgard” clade. Eubacteria are shown in teal and, at some point post-FECA, donated the mitochondrion to transitional eukaryotes via endosymbiosis. Blue designates transitional eukaryotes that led up to the LECA, which itself is the progenitor for the eukaryotic radiation and establishment of the modern recognized supergroups (see ref. 13 for more detailed discussion). (*B*) Simplified structure of the modern KT, which consists of at least 52 distinct proteins. The inner KT complex associates with variant histone marks that define the centromere and also recruit outer KT proteins. The outer KT, in turn, interacts with the spindle. (*C*) One of several routes for the evolution of the present KT, in part based on Tromer et al. (3). Early in the process of eukaryogenesis, a specific region of chromatin is marked by a histone H3 variant and likely other factors. Recruitment of several proteins to this mark, which incorporate proteins bearing both domains shared with other cellular functions as well as apparent unique architectures, provides an inner KT and may have been functional for chromosomal segregation. Recruitment or duplication of inner KT proteins provides the basis for the outer KT (and possibly transfer of the chromosomal anchor). Final expansion of the KT by further duplications, and integration with kinases for coordinate regulation of assembly and function, bring the KT to its modern form. Small curled arrows indicate paralog expansions.

Variability in mitotic mechanisms stretches back to our prokaryotic ancestors, where chromosomes exhibit distinct physical arrangements within cells from different bacterial linages and which then necessitates an accommodating organization (4, 5). The *parABS* system, which is used for segregation of chromosomes in a large number of bacterial lineages, including *Caulobacter crescentus*, is far from universal, however (6, 7), and is not used by *Escherichia coli*, for example, where the *mukBEF* SMC complex operates (8, 9). There is considerable interest into how these prokaryotic and eukaryotic mechanisms evolved, including their origins and the relationships between these disparate solutions for essentially the same problem. For eukaryotes, the configuration of the system in the last eukaryotic common ancestor (LECA) is an important facet of reconstructions of eukaryote evolutionary history (Fig. 1*A*). Most importantly, there is no evidence for common descent between known bacterial chromosome segregation systems and the eukaryotic KT, suggesting an evolutionary discontinuity in the inheritance of a fundamental and universal cellular process. The LECA stands as the root for most paneukaryotic phylogenetic reconstructions and represents a conceptual organism or, as some have suggested, population (10, 11). The LECA existed some 1.5 billion years ago, and most reconstructions suggest a surprisingly complex cellular state and plan that is essentially modern (12). Indeed, LECA compartmental complexity exceeds many extant organisms, including *Saccharomyces cerevisiae*. Regardless, the LECA cell(s) possessed a set of structures that indicate a nucleus with pretty much the same organization as in modern eukaryotes and which needed to faithfully separate chromosomes (13).

The KT consists of at least 3 subcomplexes: several variant histones and distinct inner and outer KT complexes (Fig. 1*B*). The sheer complexity of the KT, the widely disparate architectures of known KT components, and the components’ apparent interdependence have made understanding how the KT arose difficult to reconstruct, and stands in contrast to other cellular structures of similar complexity, such as the NPC and intraflagellar transport (IFT) systems in which a considerable number of subunits have obvious shared architecture. The NPC, IFT, and other systems are united in possessing multiple β−α coatomer subunits (13, 14) and have provided a framework for reconstructing the evolution of multiple endomembrane systems. In brief, these models have reached a consensus that much eukaryotic cellular architecture arose by the paralogous expansion of β−α coatomer family proteins (together with several other protein families), which diversified and led to the establishment of the NPC and IFT systems as well as antero- and retrograde vesicular transport. Critically, all of this occurred during the transition between the first eukaryotic common ancestor (FECA) and the LECA, and was complete before the LECA. Moreover, some of these factors, such as guanine nucleotide-binding proteins and proteins ancestral to the β−α architecture, may have been present in the Asgard archaea (15), considered by some as the nearest prokaryotic ancestors to the eukaryotic lineage. While the status of Asgardian genomes, which are based solely on metagenomic analysis at present, has become somewhat controversial of late, such a pathway is probably the most parsimonious interpretation of the origins of the FECA (16–18). Regardless of these events, several cellular systems, including the KT, do not obviously fit within this schema and, consequently, KT origins have been cryptic.

In PNAS, Tromer et al. (3) suggest a complex mosaic origin for the KT and posit connections with several additional cellular systems. The authors used methods that are highly sensitive for the detection of even distant relationships and which included identification of possible common protein folds that are frequently more conserved than sequence alone. Perhaps most significant is that these data suggest a mechanism by which the KT became so complex. The authors also establish that a modern KT was present in the LECA and thus must have arisen during the transition between the FECA and the LECA (Fig. 1*C*).

The KT reconstruction in Tromer et al. (3) leads to a complex containing at least 52 distinct proteins. The KT contains a cohort of proteins with relationships to other eukaryotic protein families; for example, the RWD-like family is related to E2 ubiquitin ligases as well as 5 variant histones that provide the link between the KT and chromatin. For the former family, there are potential links to the mediator complex, and several subdomains may also have a structural relationship with coatomer and TATA box-binding proteins (albeit rather limited in scope), providing links to vesicular transport and transcriptional systems. Further, there are a number of HORMA domain proteins, including the Mad2 and p31 KT components; significantly, the HORMA domain is also present in the Archaea, not only providing a prokaryotic component to the KT but also, as the HORMA KT components are monophyletic, indicating expansion of a single archaeal HORMA domain-containing gene that gives rise to the entire KT HORMA complement. Such paralog expansions are also clear for the RWD proteins and histones and provide a mechanism for generating over half of the LECA KT protein complement. One further family of proteins, the calponin homology proteins Ndc80 and Nuf2 (important in microtubule interactions), are likely eukaryotic specific, while a final group of proteins, including CenpK, CenpH, and Shugosin, have no obvious homology to other sequences in the databases (although, of course, they must have some ancestor and are perhaps also as likely to have simply diverged beyond the point of recognition). Overall, Tromer et al. (3) suggest that the KT has been assembled from a mixture of ancient, prokaryotic progenitors, with possible components from other nascent eukaryotic cellular systems, together with the presence of highly divergent or unique proteins, and is all tied together and integrated by the inevitable cohort of kinases, including the central mitotic regulator Aurora kinase.

A complement of 52 proteins means that, in terms of subunit diversity, the KT rivals the NPC and IFT systems, which also achieved their present state in the FECA-to-LECA transition and likely derived, at least in part, from Archaeal ancestral genes. Hence, the KT and other cellular systems conform to a similar overall mechanism of evolutionary origin, with paralog expansions and neofunctionalization providing much of the increased complexity. The KT does stand out from these other complexes in the heterogeneity of the architectures of its components and thus their origins. This is an important advance and provides a framework for further investigation, but it should be noted that KT diversity may well exceed the KT diversity Tromer et al. (3) report. Specifically, the searches are based around animal and fungal sequences such that any lineage-specific components that are not present in these taxa cannot have been sampled, and the overall architecture of the KT in many lineages remains unknown. For example, the recent demonstration of divergent structures for the NPCs among mammals, fungi, and plants are based around an altered copy number of components, but not changes in the complement of components themselves. This mode of highly significant change in architecture is completely silent to in silico analysis. Further, we have a highly diverse example of KT structure in the trypanosomes, a group of protozoa that likely branched from the main eukaryotic lineage quite rapidly after the LECA (19–21). Here, the entire KT bears little or no obvious resemblance to the LECA structure Tromer et al. (3) describe, which begs the question of how the LECA KT was replaced by the trypanosome form. More surprises certainly await in uncovering the origins and modifications of KTs—an exciting challenge to understanding fundamental mechanisms of genome inheritance and origins of the eukaryotic cell.
